# Loss of Function in Dopamine D3 Receptor Attenuates Left Ventricular Cardiac Fibroblast Migration and Proliferation *in vitro*

**DOI:** 10.3389/fcvm.2021.732282

**Published:** 2021-10-11

**Authors:** Andrew Kisling, Shannon Byrne, Rohan U. Parekh, Deepthy Melit-Thomas, Lisandra E. de Castro Brás, Robert M. Lust, Stefan Clemens, Srinivas Sriramula, Laxmansa C. Katwa

**Affiliations:** ^1^Department of Physiology, Brody School of Medicine at East Carolina University, Greenville, NC, United States; ^2^Department of Pharmacology and Toxicology, Brody School of Medicine at East Carolina University, Greenville, NC, United States; ^3^Department of Cardiovascular Sciences, Brody School of Medicine at East Carolina University, Greenville, NC, United States

**Keywords:** cardiac fibroblasts, dopamine D3 receptor, dopamine D1 receptor, proliferation, migration, scratch injury, D3R agonist, D3R antagonist

## Abstract

Evidence suggests the existence of an intracardiac dopaminergic system that plays a pivotal role in regulating cardiac function and fibrosis through G-protein coupled receptors, particularly mediated by dopamine receptor 3 (D3R). However, the expression of dopamine receptors in cardiac tissue and their role in cardiac fibroblast function is unclear. In this brief report, first we determined expression of D1R and D3R both in left ventricle (LV) tissue and fibroblasts. Then, we explored the role of D3R in the proliferation and migration of fibroblast cell cultures using both genetic and pharmaceutical approaches; specifically, we compared cardiac fibroblasts isolated from LV of wild type (WT) and D3R knockout (D3KO) mice in response to D3R-specific pharmacological agents. Finally, we determined if loss of D3R function could significantly alter LV fibroblast expression of collagen types I (Col1a1) and III (Col3a1). Cardiac fibroblast proliferation was attenuated in D3KO cells, mimicking the behavior of WT cardiac fibroblasts treated with D3R antagonist. In response to scratch injury, WT cardiac fibroblasts treated with the D3R agonist, pramipexole, displayed enhanced migration compared to control WT and D3KO cells. Loss of function in D3R resulted in attenuation of both proliferation and migration in response to scratch injury, and significantly increased the expression of Col3a1 in LV fibroblasts. These findings suggest that D3R may mediate cardiac fibroblast function during the wound healing response. To our knowledge this is the first report of D3R's expression and functional significance directly in mouse cardiac fibroblasts.

## Introduction

The catecholamine dopamine is heavily involved in a multitude of neural pathways of the brain. Many of these pathways involve regulating body movement, motivation, behavior, and cognitive function (1). Subsequently, dopaminergic transmission has been implicated in playing a key role in neuropsychiatric disorders such as attention deficit hyperactive disorder (2), Tourette Syndrome (3), depression (4), and neurodegenerative disorders such as Parkinson's disease (5), Huntington's disease (6), and multiple sclerosis (7, 8). However, dopamine also regulates many physiological functions in the periphery, including sympathetic output, kidney function, cardiovascular functions, and olfactory senses (7).

The biological actions of dopamine occur through five different dopamine receptors classified into two groups, D1-like or D2-like dopamine receptors (7, 9). The class of D1-like dopamine receptors include dopamine D1 and D5 receptors (D1R and D5R), while the class of D2-like dopamine receptors include dopamine D2, D3, and D4 receptors (D2R, D3R, and D4R). These dopamine receptors are G-protein coupled, with the class of D1-like dopamine receptors typically coupled to G stimulatory sites, G_s/q/olf_, to activate adenylyl cyclase, which results in increased levels of the second messenger molecule, 3',5'-cyclic-adenosine monophosphate (cAMP). Conversely, the class of D2-like dopamine receptors typically couple G inhibitory sites (G_i/o_) to adenylyl cyclase, resulting in decreased levels of cAMP (7, 9).

Since the 1980s dopamine has been implicated to play a role in hypertension (10–13). More specifically, evidence has shown that dysfunction in the D3R signaling system is associated with an increase in blood pressure in mice (14, 15). Emerging evidence suggests the existence of an intracardiac dopaminergic system plays a role in regulating cardiac function, mainly mediated by the dopamine receptor subtypes D1 and D3 (16, 17). In particular, the global loss of D3R function has been shown to negatively impact cardiac function and exacerbate the progression of fibrosis in mice (16). In addition, we demonstrated that treatment with a D3R agonist prevented morphine-induced cardiac fibrosis (18). Recently, D1R was reported to be upregulated in lung myofibroblasts of patients with idiopathic pulmonary fibrosis (19). Together, these data indicate a role for D1R and D3R in cardiac fibrosis. However, to our knowledge no studies have pursued the expression of dopamine receptors in mouse heart and their function in cardiac fibroblasts. Accordingly, this study aimed to investigate whether dopamine receptors D1 and D3 are expressed both in mouse cardiac tissue and primary cultures of cardiac fibroblasts and explored the potential role of D3R in cardiac fibroblast function.

## Materials and Methods

### Animals

All animal experimental procedures were approved by the Institutional Animal Care and Use Committee at East Carolina University and followed National Institute for Health guidelines outlining animal care and use in a laboratory setting (The Guide-NRC 2011; 8th edition). All efforts were made to minimize the number of animals used. We used 3–6-month-old, male and female wild-type (WT) mice (C57BL/6J, *n* = 20), and dopamine D3 receptor global knockout mice (D3KO; strain B6.129S4-Drd3^tm1dac^/J, stock # 2958; *n* = 20). The D3KO mice were obtained from Jackson Laboratory, Bar Harbor, ME and maintained as a breeding colony at ECU.

### Cardiac Fibroblast Cell Isolation

The left ventricles were removed from whole hearts of WT and D3KO mice for cell culture. Tissues were first washed with ice cold 1x PBS (Invitrogen; AM9624) to remove any remaining blood, minced, and then digested with collagenase II (Worthington Biochemical Corp.; 46A034) containing DNaseI (Worthington Biochemical Corp.; LS002139) at 37°C for 15 min. Next, the digested tissues underwent two rounds of centrifugation (300 xg for 7 min each) with removal of supernatant each time to discard any debris remaining from the collagenase digestion. Finally, cell pellets were suspended in 1x DMEM/F-12 (1:1) media (Gibco; 11320-033) supplemented with 10% fetal bovine serum (FBS) (Gibco; 10438-026) and incubated at 37°C + 5% CO_2_ in T25 cell culture flasks until fibroblasts were firmly attached. After the cells were attached, the flasks were washed in warmed 1x PBS to remove debris and placed back into the incubator and allowed to grow to 70% confluency before subculture. Primary cultures were identified and confirmed as cardiac fibroblasts by spindle-shaped morphological appearance and vimentin staining.

### Immunofluorescence Labeling of D1R and D3R in Heart Tissue and Primary Cardiac Fibroblasts

Hearts were excised from WT animals, rinsed with saline, immediately inserted into OCT compound block molds, and placed on dry ice for ~30 min before being stored in a −80°C freezer. The cryosections of 10 microns thickness were positioned on glass microscope slides and fixed in 4% paraformaldehyde for 15 min at room temperature. Similarly, primary cultures of cardiac fibroblasts from the LV of WT and D3KO animals were seeded (5,000 cells/well) and grown on 0.2% gelatin coated glass coverslips in a 24-well plate and then fixed with 4% paraformaldehyde in PBS for 15 min at room temperature. Cells were permeabilized with 0.1% Triton X-100 in PBS for 10 min and blocked in 1% bovine serum albumin (BSA) in 1x PBS containing 0.3% Tween 20 for 1 h at room temperature. Both cells and tissues were then incubated with primary antibodies for Vimentin [M0725, lot #027(102), Dako, 1:500 dilution], D1R (NB110-60017AF488, lot #B-3-101620, Novus Biologics, 1:500 dilution), and D3R (bs-1743R-Cy5, lot #AF12125641, Bioss, 1:500 dilution) overnight at 4°C. Both cells and heart tissue were then washed the next day 3 times with 1x PBS containing 0.3% Tween 20. Then they were incubated with secondary antibodies (Donkey anti-Mouse Alexa Fluor Plus 647, 1:1000 dilution or Donkey anti-Mouse Alexa Fluor Plus 555, 1:1000 dilution) for 1 h at room temperature. Following 3 additional washes with 1x PBS containing 0.3% Tween 20 for 15 min, cells and heart tissue were then placed on slides and covered with coverslips using ProLong Diamond antifade reagent with DAPI (ThermoFisher). The images were captured with an Echo Revolve microscope.

### Western Blot Analysis for D1R and D3R in Heart Tissue and Primary Cardiac Fibroblasts Isolated From LV

C57BL/6 mice (Charles River stock #027), male adults (*n* = 6 males), were euthanized with an overdose of inhalational isoflurane and the heart removed as per the protocol approved by the Institutional Animal Use and Care Committee. LV was isolated and snap frozen in liquid nitrogen. The protein from the LV tissues and confluent cardiac fibroblasts were isolated using RIPA buffer with 1x protease and phosphatase inhibitors and protein was quantified by Bradford assay. Western blots were performed by loading 10 μg of protein into a 4–20% Bio-Rad precast gel. After electrophoresis, proteins were transferred and immunoblotting was performed for dopamine receptors: D1R (1:500, ab78021 Abcam); and D3R (1:5000, ab42114 Abcam) (20). Image acquisition was performed using a UVP ChemiDoc-It TS2 Imager.

### Cardiac Fibroblast Proliferation Rate

Equal number of sub-confluent cardiac fibroblast cells were seeded in 24-well plates and placed in the incubator at 37°C and 5% CO_2_ for 36 h to allow cells to attach. The old growth media was discarded, and cells were washed with 1x PBS and supplemented with fresh media with treatments. Stock solutions of the agonist and antagonist were prepared in DMSO as per manufacturer guidelines and were diluted accordingly. Treatments were performed in triplicates for both D3KO and WT cardiac fibroblasts as follows: (1) Vehicle control (DMEM + 10% FBS + 0.1% DMSO); (2) Pramipexole [10μM] (Tocris Bioscience), an agonist specific for D3R; (3) SB-277011-A (SB) [10μM] (Tocris Bioscience), an antagonist specific for D3R. Addition of treatments indicated the start of the experiment and Pramipexole and SB were replenished every 12 h. At the time points of 6, 12, 24, and 36 h, the respective 24-well plates were removed from the cell culture incubator and treated with 0.05% Trypsin-EDTA (1x) (Gibco™; 15400-054) to dissociate the cells. Once cells were detached, an equal volume of Trypsin Neutralizer (1x) (Gibco™; R-002-100) was added and cell counts of each treatment were recorded for all samples at different time points of 6, 12, 24, and 36 h, respectively. Cell counts were achieved using 0.4% Trypan Blue staining in conjunction with the Countess II Automated Cell Counter (Invitrogen; AMQAX10000).

### Cardiac Fibroblast Migration in Response to Scratch Injury

WT and D3KO cardiac fibroblast cells were seeded on 6-well plates at a count large enough to ensure ≥60% confluency upon start of the experiment and placed in the incubator at 37°C and 5% CO_2_ for 36 h to allow cells to adhere. Wells were then washed with 1x PBS prior to making scratches and adding treatments. Next, three parallel scratches were made in each well-designated for scratches using Falcon® Cell Scrapers, creating a total of nine scratches (*n* = 9) for each treatment group at each time point. Drug treatments were diluted in DMEM/F12 containing low FBS (0.5%) to facilitate serum-starved conditions and limit the effect of cell proliferation on scratch closure. Treatment groups were added in duplicates and were mirrored for both D3KO and WT cardiac fibroblast cells as follows: (1) Vehicle control (DMEM + 0.1% DMSO); (2) Pramipexole [10μM] (Tocris Bioscience), an agonist specific for D3R; **3)** SB-277011-A (SB) [10μM] (Tocris Bioscience), an antagonist specific for D3R. Upon initiation of scratches and treatments, pictures were taken of the initial scratches and the cells were placed in the incubator at 37°C + 5% CO_2_. Additional pictures were taken at the respective time points (3, 6, 12, and 24 h) to assess progression of gap closure under each treatment. Images of scratches were measured for cell migration distance (μm) at specific time points using the software ImageJ, installed from https://nih.gov.

### Real Time-Quantitative PCR

Hearts were excised from WT and D3KO animals and snap frozen in liquid nitrogen. Frozen mouse heart tissue samples (*n* = 3) were homogenized in TRIzol reagent and confluent cardiac fibroblasts were lysed and suspended in TRIzol reagent (15596-026). RNA was isolated from mouse heart tissue and primary cardiac fibroblasts using TRI/Direct-Zol RNA Miniprep Kit (Zymo Research; R2072). A cDNA library of all samples was created using Superscript IV reverse transcriptase with ezDNAse enzyme (Invitrogen; 11766050) according to manufacturer's instruction (Pub. No. MAN0015862). A volume of 1μL of cDNA from each sample was added to the respective wells of a 384-well DNase/RNase-free PCR plate as well as the necessary reagents for RT-qPCR. Thus, each well-included materials as follows: 0.5μL primer for specified gene, 0.5μL β-actin (housekeeping gene), 4μL TaqMan™ Fast Advanced Master Mix (Applied Biosystems, 4444557), 1μL cDNA, and 4μL DNase/RNase-free ultrapure water (Invitrogen, 10977023) for 10μL total volume in each well. Samples were added in duplicates. TaqMan primers were acquired from Applied Biosystems (Life Technologies, ThermoFisher Scientific) and are as follows with assay IDs: *Drd1* (Mm02620146_s1), *Drd3* (Mm00432887_m1), *Col1a1* (Mm00801666_g1), *Col3a1* (Mm00802296_g1) and *ACTB* (Mm02619580_g1). Quantitative real-time-PCR was performed using Applied Biosystems QuantStudio 6 Flex System at manufacturer's recommended settings. C_T_ values from QuantStudio program were then transformed to relative gene expression values by the comparative C_T_ method (21, 22).

### Statistics

Analysis of Variance (ANOVA) was performed on proliferation and migration mean values to determine if there was statistical significance between 3+ sample means for each time point. ANOVA was followed by two-tail, two sample Student *t*-tests at time points that resulted in ANOVA *p*-values less than or equal to 0.05. Two-tail, two sample Student *t*-tests were also performed on all real-time PCR data, with *p*-values less than or equal to 0.05 determined to be statistically significant using GraphPad Prism.

## Results

### Heart Tissue and Cardiac Fibroblasts Express Dopamine Receptors D1R and D3R

**Figure 1:**
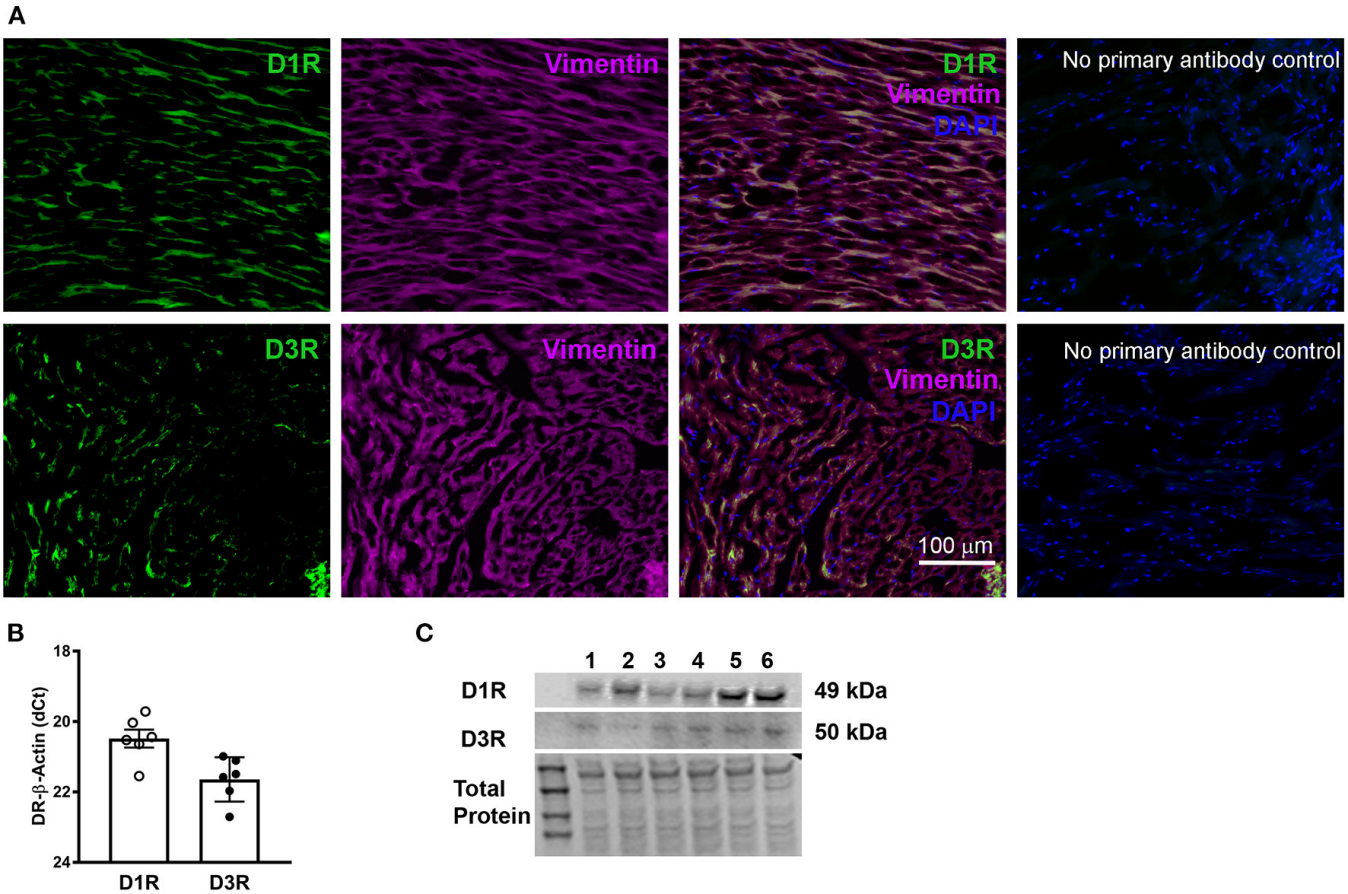
The expression and distribution of dopamine receptors, D1R and D3R in mouse hearts: **(A)** Representative photomicrographs showing immunofluorescence staining of D1R and D3R which co-localized with vimentin, a cardiac fibroblast-specific marker, in the heart tissue sections. No primary antibody control sections were incubated with the antibody diluent alone and no primary antibody, followed by incubation with secondary antibodies and DAPI (the nuclear stain), which confirms the antibody specificity of D1R and D3R. **(B)** Gene expression was determined using TaqMan primers by real-time qPCR. The normalized -dCT values indicate that there is relatively higher expression of D1R compared to D3R in cardiac tissue. **(C)** Receptor protein expression determined by western blot using receptor specific antibodies as described in methods section.

We first confirmed the expression of D1R and D3R within the hearts of WT mice. Immunofluorescence labeling using specific antibodies against D1R and D3R revealed that both receptors are expressed in mouse LV **(**Figure 1A**)**. Furthermore, the co-localization of D1R and D3R with the fibroblast marker vimentin, confirmed the expression of these receptors in the cardiac fibroblasts **(**Figure 1A**)**. In addition, gene and protein quantification confirmed the expression of both D1R and D3R in the mouse hearts **(**Figures 1B,C**)**. The data clearly demonstrate that D1R and D3R express at mRNA and protein levels in mouse hearts.

**Figure 2:**
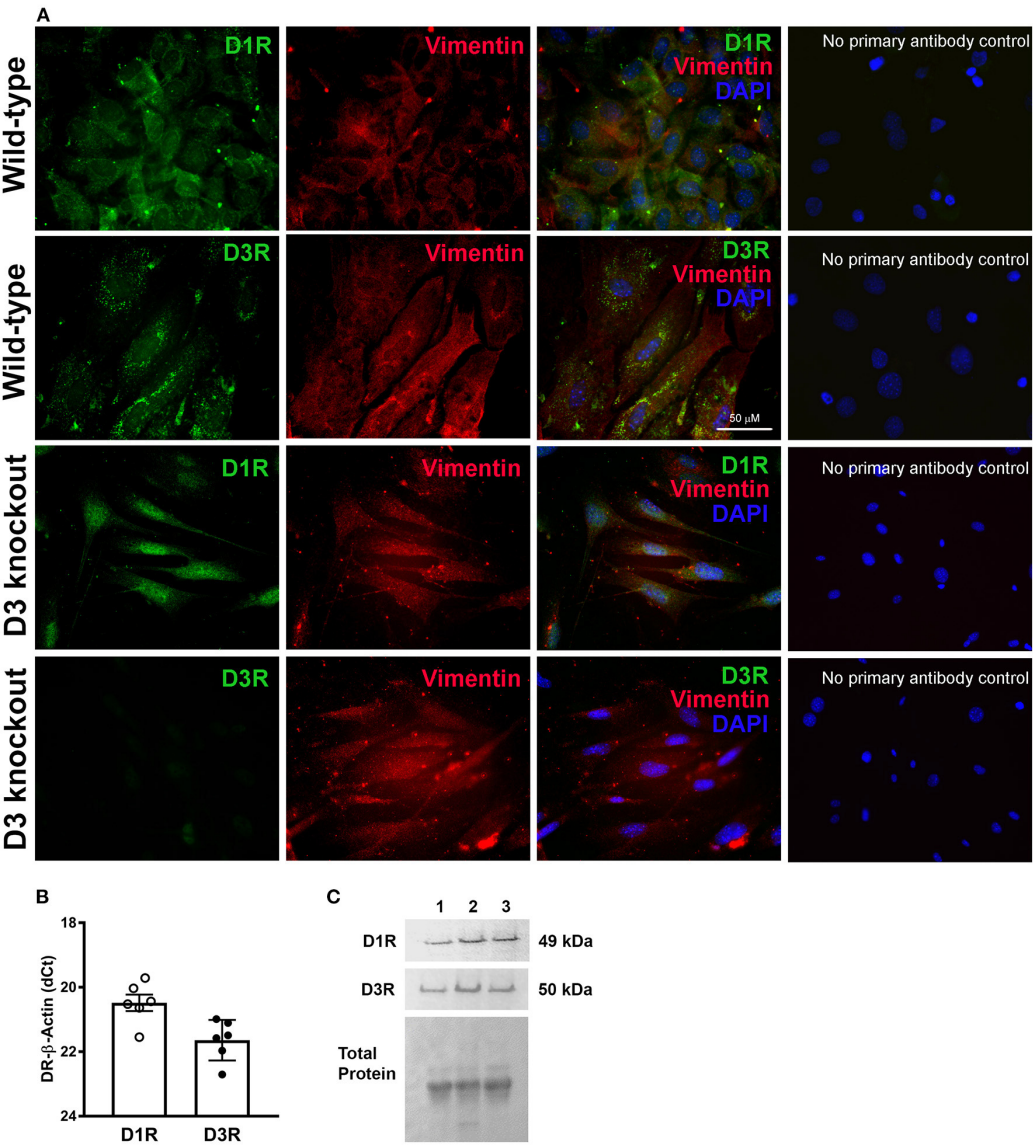
The expression and distribution of dopamine receptors, D1R and D3R in mouse primary cardiac fibroblasts: **(A)** Representative photomicrographs showing immunofluorescence staining of D1R and D3R which co-localized with cardiac fibroblasts specific marker vimentin staining in the isolated WT and D3KO primary cardiac fibroblasts in culture. Cardiac fibroblast expression of D1R and D3R is not lost when primary cells are maintained in cell culture. As expected, there is no observable staining for D3R in D3KO cardiac fibroblasts. No primary antibody control images show the specificity of the primary antibodies used. **(B)** Gene expression determined using TaqMan primers by real-time qPCR. The normalized -dCT values indicate that there is relatively higher expression D1R compared to D3R in cardiac fibroblasts. **(C)** Receptor protein expression determined by western blot using receptor specific antibodies as described in methods section.

Furthermore, we confirmed the expression of D1R and D3R in isolated mouse heart primary cultures of LV cardiac fibroblasts from WT and D3KO mice. As indicated by co-localization of receptor specific immunofluorescence with the fibroblast marker vimentin, both D1R and D3R are expressed in mouse cardiac fibroblasts, and the absence of D3R staining in D3KO reflected the positive functional knockout for this model **(**Figure 2A**)**. In addition, gene **(**Figure 2B**)** and protein **(**Figure 2C**)** analyses confirmed the expression of both D1R and D3R in the primary cultures of cardiac fibroblasts.

### D3R Deletion or Dysfunction Reduce Cardiac Fibroblast Proliferation and Migration

**Figure 3:**
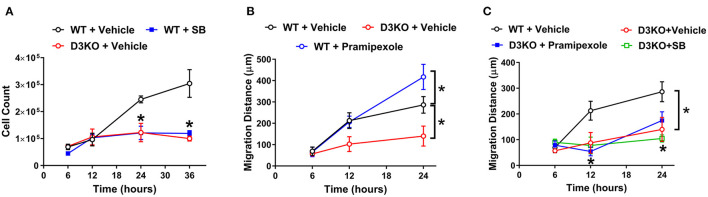
Cardiac fibroblast cell proliferation and migration studies: **(A)** Cardiac fibroblast cell proliferation between WT + vehicle (black circle), WT treated with SB (closed blue square), and D3KO + vehicle (red circle) over a time-period of 36 h. Cells were incubated in DMEM/F-12 growth media supplemented with 10% FBS; *n* = 3 for each treatment at each time-point, ^*^p < 0.05 statistically significant. Bars represent standard error of mean. **(B)** Cardiac fibroblast cell migration distance in response to a scratch injury over a 24-h time-period. Migration distance in μm between WT + vehicle (black circle), D3KO + vehicle (red circle), and WT cells treated with D3R specific agonist, pramipexole (blue circle). Cells were incubated in DMEM/F-12 growth media supplemented with 0.5% FBS; *n* = 9 for each treatment at each time-point, ^*^p < 0.05 statistically significant. Bars represent standard error of mean. Statistical significance between WT treatment and D3KO + vehicle. **(C)** Cardiac fibroblast cell migration distance in response to a scratch injury over a 24-h time-period. Migration distance in μm of D3KO + vehicle cells (red circle), D3KO cells treated with pramipexole (closed blue square), and D3KO cell treated with SB (green square), compared against WT + vehicle cells (black circle); *n* = 9 for each treatment at each time-point, ^*^p < 0.05 statistically significant. Bars represent standard error of mean.

To determine whether D3R loss of function may play a role in cardiac fibroblast processes, we compared proliferation and migration rates between WT and D3KO cells. Cardiac fibroblast proliferation differed over a period of 36 h between WT and D3KO controls, with significant differences (*p* < 0.05) occurring at 24 and 36 h (Figure 3A). Between 6 and 12 h, the proliferation rates of D3KO and WT cells are comparable; however, while the proliferation of the WT cells steadily increased until 36 h, no further change was observed in the D3KO cells. Compared to WT, the proliferation of D3KO fibroblasts significantly decreased after 24 h (2-fold) and 36 h (3-fold) (Figure 3A). The data indicate that D3R dysfunction could cause a decrease in the rate of fibroblast proliferation. Next we utilized a pharmacological approach to confirm the role of D3R in WT cardiac fibroblast proliferation. Treatment with a D3R antagonist, SB-277011-A (SB), resulted in WT cardiac fibroblast proliferation that behaved similarly to that of D3KO + vehicle cells (Figure 3A, blue line). This result further demonstrates the importance of functional D3R on the rate of proliferation.

The migratory response of cardiac fibroblast cells is a key element in the wound healing of the heart. Thus, we next evaluated whether there was a difference in cell migration in response to a scratch injury between WT and D3KO cells. Figure 3B shows the difference in migration rates over 24 h between WT + vehicle and D3KO + vehicle cells, as well as WT cells treated with pramipexole, a D3R agonist. There was no significant difference in the migration rate between cell types throughout the initial 12 h (Figure 3B). However, at 24 h post-scratch, WT + vehicle cells and WT + pramipexole cells had covered a significantly greater distance than D3KO + vehicle cells (~270 μm more in the WT + pramipexole cells, and ~145 μm more in the WT + vehicle cells) (Figure 3B). Treatment with D3R agonist, pramipexole, resulted in an enhanced rate of migration of WT cells after 24 h when compared to WT + vehicle cells (~400 μm total in WT + pramipexole and ~275 μm total in WT + vehicle, *p* < 0.05) (Figure 3B). The stimulation of D3R resulted in an increased migration rate, implying that active signaling through D3R could be essential in cardiac wound healing.

Finally, to confirm that pharmacological treatments have no significant influence on the knockout model, D3KO cardiac fibroblasts were treated with either pramipexole or SB during the migration assay. When treated with pramipexole, D3KO cells appeared to have a greater rate of migration after 12 h when compared to the other D3KO groups (Figure 3C). However, it was observed that treatment of D3KO cardiac fibroblasts with either pramipexole or SB did not result in any significant change in migration distance over the 24-h period when compared to D3KO control cells (Figure 3C). The lack of cellular response to pramipexole and SB indicates that the D3R within the knockout model are indeed non-functioning.

### Expression of Col1a1 and Col3a1in Heart Tissue and LV Cardiac Fibroblasts of WT and D3KO

**Figure 4:**
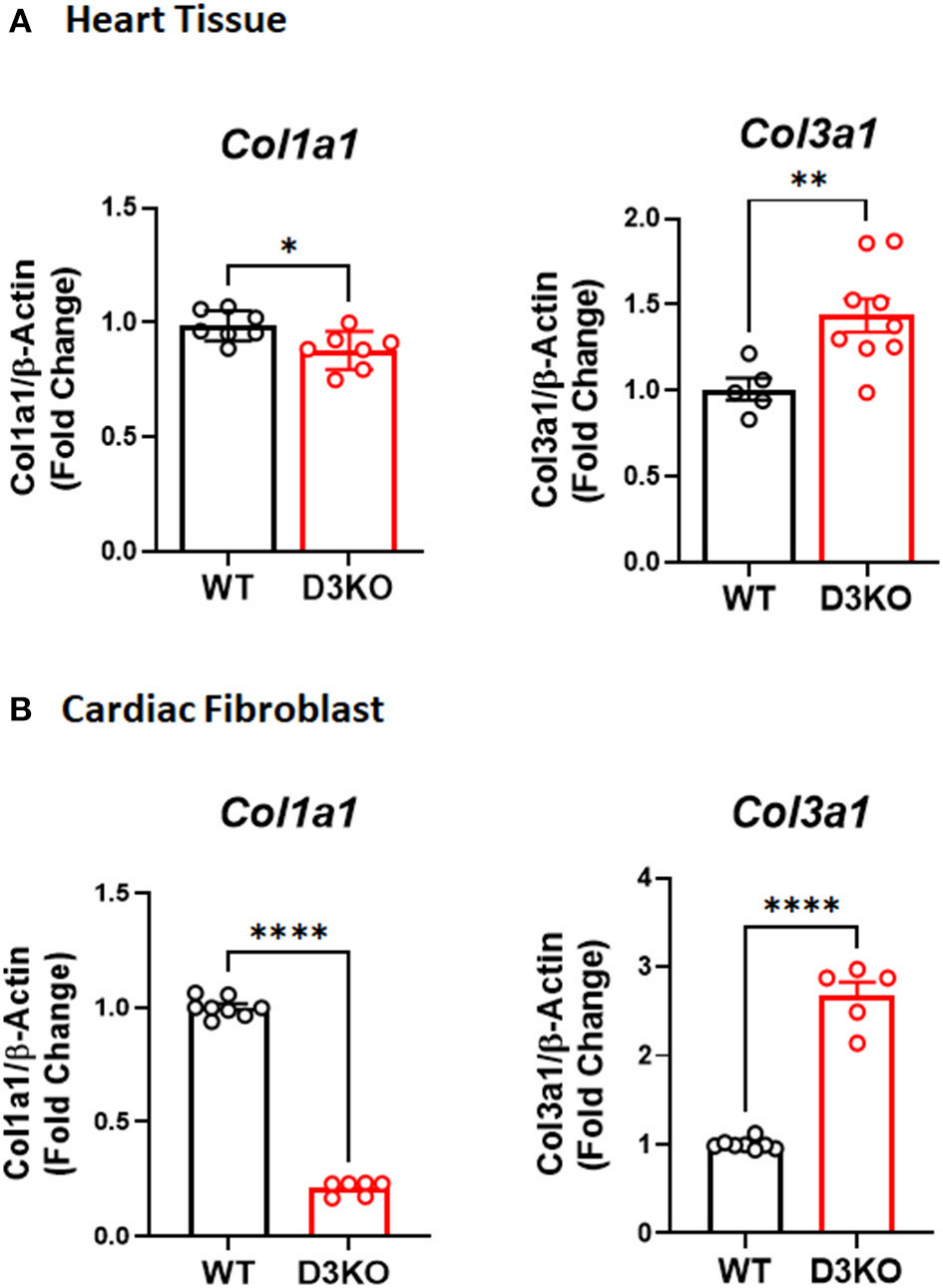
Collagen Gene Expression in WT and D3KO Cardiac Tissue and Fibroblast Cells: **(A)** Gene expression determined using TaqMan primers by real-time qPCR (*n* = 3, run in duplicates); Gene expression data for Col1a1 and Col3a1 was derived from WT and D3KO whole heart tissues. All values are Fold Change normalized to WT. There is a significant decrease in Col1a1 gene expression in D3KO heart tissue compared to WT and a significant increase in Col3a1 gene expression in D3KO heart tissue compared to WT. ^*^p < 0.05, ^**^p < 0.01, statistically significant; Bars represent standard error of mean. **(B)** Gene expression determined using TaqMan primers by real-time qPCR (*n* = 4, run in duplicates); Gene expression data for Col1a1 and Col3a1 was derived from WT and D3KO LV cardiac fibroblasts. All values are Fold Change normalized to WT, ^****^p < 0.0001 statistically significant, bars represent standard error of mean.

A predominant function of cardiac fibroblast is to secrete type I and type III collagens. As such, we investigated whether loss of D3R also influences cardiac collagen expression. Our gene expression results indicate that the expression of both Col1a1 and Col3a1 were quite different between D3KO and WT hearts, as Col1a1 was significantly decreased and Col3a1 was significantly increased in D3KO compared to WT (Figure 4A). Next, we analyzed Col1a1 and Col3a1 gene expression in WT and D3KO LV cardiac fibroblasts, the results of which showed a similar trend as in the cardiac tissue (Figure 4B).

## Discussion

This is the first study to demonstrate that the D3 receptor is present in mouse cardiac tissue and LV fibroblasts, and that it has an influence on cardiac fibroblast proliferation, migration, and collagen expression. While much of the literature describes D3R's role in tissues such as the brain (23, 24), kidney (25, 26), and vasculature (27–29), a previous study inquired about the influence of D3R in the aging heart (16). Several reports have shown direct interaction between D3R and D1R (30–32); thus, this study focused on both receptors and aimed to confirm the expression of D1R and D3R in mouse cardiac tissues and cardiac fibroblasts in addition to investigating whether D3R can affect multiple cardiac fibroblast functions.

Confirmation of the expression of D1R and D3R within WT mouse cardiac fibroblasts and heart tissue is essential due to lack of comprehensive analysis in the existing literature. Figure 1 shows LV tissue expresses both D1R and D3R; although, expression of these receptors was not exclusive to cardiac fibroblasts since expression was also observed in vimentin negative cells. Likewise, Cai and colleagues have demonstrated expression of D1R and D2R in cultured neonatal rat cardiomyocytes (33), which may suggest the possibility that D1R and D3R are also expressed in mouse adult cardiomyocytes. Not surprisingly, previous studies have shown that both receptors are expressed in mouse brain and spinal cord (7, 34, 35). Yet, other studies have observed D1R RNA in fibroblasts isolated from both human and mouse lung tissue (36). So, in addition to other organ systems, D1R and D3R are expressed in mouse cardiac fibroblasts, demonstrating expression of these receptors across multiple different tissue types.

Use of non-ergot dopamine agonists is common among human patients with Restless Legs Syndrome (RLS) (37–39) and Parkinson's disease (40–44). Though these agonists do not present the potential for heart valve regurgitation that some ergoline-derived agonists do (44, 45), they can still lose efficacy over prolonged use (46, 47). To investigate whether manipulation of D3R affects cardiac fibroblast function, we utilized D3R-specific agonist and antagonist, pramipexole and SB, respectively, as well as a cardiac fibroblast D3KO model. Our proliferation results indicate that there is a noticeable attenuation of fibroblast proliferation upon loss of functional D3R (Figure 3A). Additionally, it was observed that treatment with SB resulted in WT cardiac fibroblast proliferation behavior similar to the proliferation seen in D3KO + vehicle fibroblasts (Figure 3A). Interestingly, this decrease in proliferation was accompanied by a definite decrease in cell viability over the 36-h time-period in both D3KO + vehicle and WT + SB (Figure 3A). A possible reason for these observations could be that D3R modulates an apoptotic pathway in cardiac fibroblasts. Previous work has shown that stimulation of D3R has a protective effect against apoptosis in neural cells through inhibition of JNK and caspase pathways and increased levels of bcl-2 and pAkt (48). Additionally, stimulation of D3R has been demonstrated to reduce kidney epithelial cell apoptosis, likely through inhibition of Gα_12_ (49). Also, studies have shown that D3R positively modulates the proliferation of cells of different brain regions, either by stimulation of the receptor, as reported by a study regarding neural cells of the subventricular zone (50) or by inhibiting the receptor, as a study reported regarding cells of the hippocampus (51). Furthermore (52), found that knockdown of D3R results in cytokinesis defects in HeLa cells (52).

Complementing the decreased proliferation observed in vehicle-treated D3KO cardiac fibroblast cells was the observation of attenuated migration in this same cardiac fibroblast model (Figures 3B,C). Of note, decreased migration rate in D3KO cells may be a cumulative effect of reduced migration and proliferation. This potentially falls in line with the results from a study performed by Yasunari et al. (53), wherein a specific D1-like antagonist reversed the antimigratory effects of dopamine in vascular smooth muscle cells (53). Since the two families of dopamine receptors, D1-like and D2-like, are separated based on each family's opposing influences on cAMP production (1), it could be inferred that they also have opposite influences on cell migration. While some studies propose D1-like receptors are antimigratory and antiproliferative in vascular smooth muscle (53), other studies propose that D2-like receptors are also antiproliferative (54) and antimigratory (55). Furthermore, interaction between D1R and D3R in vascular smooth muscle has been described (29) and could explain the antiproliferative and antimigratory effects of these receptors in this tissue type. Although these studies describe the role of the dopamine receptors in vascular smooth muscle, these receptors could affect migration differently in cardiac fibroblast cells, as was observed in our migration assay **(**Figures 3B,C**)**. Our data suggests that the D2-like receptor family is antimigratory and antiproliferative upon the knock down of D3R.

Collagen proteins are critical components of the extracellular matrix and are present in various types throughout the human body. Though with regards to the cardiac extracellular matrix, collagen types I and III predominate, with the ratio of these types serving as an important factor in cardiac physiology and disease. If the ratio is tilted too far out of balance in favor of either collagen type (I or III), then complications will arise (56). Our collagen expression data clearly indicate that there is a significant difference in basal collagen Col1a1 and Col3a1 expression in the D3KO heart, likely contributed by the significantly altered collagen I and III expression profiles of the D3KO LV cardiac fibroblasts (Figures 4A,B). Hence, this shift in collagen ratio toward increased deposition of collagen type III and reduced collagen type I may make D3KO hearts more susceptible to cardiac dysfunction and fibrosis.

Nonetheless, since detailing the mechanistic insight was beyond the scope of this brief report, certain research limitations accompany our study. For instance, the D3R-dependent mechanisms which mediate fibroblast proliferation, migration, and collagen expression remain to be established and future studies will be required to better determine the effects of D3R on cardiac remodeling and dysfunction.

## Conclusions

Fibroblasts are key mediators in the synthesis and degradation of collagen and are major regulators of the physiological and pathological fibrotic states of the heart (57). Johnson et al. previously observed a fibrotic state in 2-month-old D3KO mice comparable to that observed in 2-year-old WT mice (16). Our study investigated further into this observation to determine whether cardiac fibroblast function may be altered in this animal model. Accordingly, it was observed that inhibition or dysfunction of D3R in cardiac fibroblasts results in significantly altered collagen expression and attenuated fibroblast proliferation and migration, whereas stimulation with a D3R agonist enhances fibroblast migration in response to a scratch injury. This suggests D3R may play a novel role in the wound healing process. Therefore, the next step will be to elucidate the mechanisms involved in cellular organization that may be regulated by this receptor.

## Data Availability Statement

The original contributions presented in the study are included in the article/supplementary material, further inquiries can be directed to the corresponding author.

## Ethics Statement

The animal study was reviewed and approved by Institutional Animal Care and Use Committee at East Carolina University.

## Author Contributions

LK: conception and design. AK, SB, DM-T, RP, SS, and LK: data acquisition and analysis. LK, LC, SC, RL, and SS: data interpretation and drafting of the manuscript. AK, LK, SB, LC, SC, RL, and SS: revision of the manuscript. All authors contributed to the article and approved the submitted version.

## Funding

This study was supported, in part, by the Department of Physiology, the Brody Brothers Endowment Fund (LECB and LCK), by the National Heart, Lung, and Blood Institute of the National Institutes of Health under award number HL152297 (LECB) and R01HL153115 (SS), and the American Heart Association Grant No. 18AIREA33960311 (LECB).

## Conflict of Interest

The authors declare that the research was conducted in the absence of any commercial or financial relationships that could be construed as a potential conflict of interest.

## Publisher's Note

All claims expressed in this article are solely those of the authors and do not necessarily represent those of their affiliated organizations, or those of the publisher, the editors and the reviewers. Any product that may be evaluated in this article, or claim that may be made by its manufacturer, is not guaranteed or endorsed by the publisher.
